# Hepatitis A Virus Infection and the Waste Handling Industry: A Seroprevalence Study

**DOI:** 10.3390/ijerph9124498

**Published:** 2012-12-07

**Authors:** George Rachiotis, Dimitrios Papagiannis, Efthimios Thanasias, George Dounias, Christos Hadjichristodoulou

**Affiliations:** 1 Department of Hygiene and Epidemiology, Medical Faculty, University of Thessaly, Larissa 41222, Greece; E-Mails: gsrachmed@yahoo.com (G.R.); dpapajon@yahoo.gr (D.P.); dr.makis@gmail.com (E.T.); 2 Department of Occupational and Industrial Hygiene, National School of Public Health, Athens 115-21, Greece; E-Mail: gdounias@esdy.edu.gr

**Keywords:** hepatitis A infection, municipal solid waste workers, occupational exposure

## Abstract

Waste collectors have a theoretical risk of Hepatitis A virus infection. The aim of the study was to assess the prevalence and risk factors of hepatitis A virus infection (HAV) among municipal solid waste workers (MSWWs) in a municipality of central Greece. A seroprevalence study of HAV was conducted among 208 employees (100 waste collectors and 108 municipal gardeners) of a municipality in central Greece. Total antibodies against HAV were measured and information regarding potential risk factors was collected through a face to face interview. The prevalence of HAV infection among the municipal waste collectors was 61% *vs*. 27% among municipal gardeners. Logistic regression analysis showed that exposure to waste (OR = 2.87; 95% CI = 1.24–6.62) and age (OR = 22.57; 95% CI = 7.29–69.88) were independently associated with the anti-HAV positivity. Moreover, waste collectors who reported smoking/drinking/eating during waste collection were at higher risk of HAV infection (RR = 2.84; 95% CI = 1.73–4.63). Stratified analysis among municipal waste collectors indicated an independent association between eating/smoking/drinking during waste collection and anti-HAV (+) (OR = 3.85; 95% CI = 1.34–11.06). Occupational exposure to waste is a potential risk factor for HAV infection. Smoking/eating/drinking during waste collection could be the mode of hepatitis A virus transmission among municipal waste collectors.

## 1. Introduction

Municipal Solid Waste Workers (MSWW) are exposed to a variety of occupational hazards and the need for an evidence based Worker Health Surveillance has been pointed out [1,2,3]. However, to our knowledge, there are only a limited number of studies investigating the HAV infection among municipal solid waste workers [4,5,6,7]. There is some evidence that municipal waste collectors could be at increased risk of HAV infection. It has been suggested that the infection pathway of HAV infection among municipal waste collectors could be related to some activities which enhance the activation of the faecal-oral route (e.g., smoking, eating without adherence to universal precautions). As far as we know; these hypotheses have not been tested regarding the possible association between waste collection and risk of HAV infection.

## 2. Methods

This study is a part of a wider research project on prevalence and risk factors of viral hepatitis A and B among municipal waste collectors. Details about this project have been reported elsewhere [8]. The present study was a hepatitis A viral infection seroprevalence study of municipal workers in a big municipality of central Greece during the period 2007–2008. Municipal waste collectors (120) and municipal gardeners (150) were invited to participate in the study. One hundred and two out of 120 waste collectors participated in the study (response rate: 85%). Additionally, 111 out of 150 municipal gardeners accepted to participate (response rate: 74%). The waste collectors are responsible for the collection of household waste. The municipal gardeners are responsible for maintaining, developing and remodeling municipal gardens. Two waste collectors and three gardeners reported vaccination against hepatitis A virus infection and have been excluded from the study. The final sample of our study consisted of 100 municipal waste collectors and 108 gardeners. A questionnaire was used to gather social and demographic information and to define the extent of the workers’ exposure to occupational injuries. All employees gave their informed written consent in order to participate in the study. The questionnaire included questions about sex, age, educational status (completed years of education), job title (waste collectors, gardeners), duration of employment (continuous variable), travel to Asia (except Japan) (yes, no), shellfish consumption (often/sometimes, no, and country/region of origin. In addition, the participants were asked to report previous history of vaccination against HAV. Finally, the participants were asked about their exposure to smoking, drinking, eating during waste collection (never/almost never/rarely/sometimes/frequently/almost always). Waste collection workers who reported smoking or drinking during waste collection frequently/almost always have been considered as the “high exposure category”. Waste collection workers who reported smoking/drinking during waste collection never/almost never/rarely/sometimes have been considered as the “low exposure category”. The questionnaires were completed through a face to face interview with the participants. All sera collected were tested for total anti-HAV antibodies by enzyme linked immunosorbent assay (ELISA).

## 3. Statistical Analysis

Participants were distributed into groups according to sex (male or female), age (cut-off based on the mean age: 44 years), years of education (nine years or lower and over nine years of education) and years of employment (over or under 16 years of employment, mean duration of municipal employment: 16 years). These cut-offs were selected based on the mean of each variable included in the study. Absolute (n) and relative (%) frequencies were presented for qualitative variables, while continuous variables were presented as mean (standard deviation). Chi-square (X^2^) test and Student’s *t*-test were used for the analysis of qualitative and continuous data, respectively. A logistic regression model was used as the multivariate analysis. Age, occupational exposure to waste, education and duration of employment were the independent variables while the presence of positive anti-HAV in the workers’ serum was the dependent variable in this model. Moreover, in order to further explore the nature of the association between smoking/drinking/eating during waste collection and anti-HAV (+) we performed a stratified analysis by age, duration of exposure, and education. Odds ratio, and 95% Confidence Intervals (95% CI) were calculated. The level of statistical significance was set at 0.05. The analysis was performed by the use of SPSS (version 14.0) and Epi Info software. The protocol of the study has been approved by the scientific committee of the Postgraduate program: “Applied Public and Environmental Hygiene” of the University of Thessaly. The participants provided informed written consent.

## 4. Results

The basic socio-demographic information of the participants by exposure status is presented in Table 1.

**Table 1:** Comparative analysis of socio-demographic characteristics by exposure status among municipal workers.

Characteristic	Exposed to waste (waste collectors) (+)	Non exposed to waste (gardeners) (−)	*p* value
**Sex**	n/N, (%)	n/N, (%)	
male	89/100 (89%)	49/108 (45.3%)	<0.001^ b^
female	11/100 (11%)	59/108 (54.7%)	
**Age**^ a^	47.93 (8.8)	42.73 (7.5)	<0.001^ c^
**Duration of employment (years)** ^a^	17.11 (7.7)	13.91 (5.4)	0.001^ c^
**Educational status**			
≤9 years	69/100 (69%)	49/108 (45.4%)	0.001^ b^
>9 years	31/100 (31%)	59/108 (54.6%)	

^a^ mean, standard deviation. ^b^ X^2^ test. ^c^ Student’s *t*-test.

Municipal solid waste collectors were older, less educated and with longer duration of employment in comparison to the group of municipal gardeners.

The prevalence of HAV infection among waste collectors was 61% *vs*. 27% among the reference population (*p* < 0.001). Univariate analysis (Table 2) shows that occupational exposure to waste, age, education, duration of employment and sex were significantly associated with the prevalence of HAV infection. Logistic regression analysis (Table 3) indicated that age (OR = 22.57; 95% CI = 7.29–69.88), occupational exposure to waste (OR = 2.87; 95% CI = 1.24–6.62), duration of employment (3.57; 95% CI = 1.15–11.08) and education (OR = 2.19; 95% CI = 1.01–4.78) were independently associated with the risk of HAV infection. Further statistical analyses among waste collection workers suggest that waste collectors who reported smoking or eating/drinking during waste collection had a higher prevalence of HAV (80% *vs*. 20% for waste collection workers who did not report smoking/drinking or eating during waste collection (Table 4). In particular municipal waste collection workers who exposed to smoking/drinking/eating during waste collection had an increased risk of HAV infection (RR = 2.84; 95% CI = 1.73–4.63). Moreover, stratified analysis among municipal waste collectors indicated an independent association between eating/smoking/drinking during waste collection and anti-HAV (+), after controlling for age (OR = 3.85; 95% CI = 1.34–11.06), duration of employment (OR = 6.07; 95% CI = 1.02–6.46) and education (OR = 3.83; 95% CI = 1.1–6.38). These figures suggest that municipal waste collectors that didn’t report exposure to poor working practices such as eating/drinking/smoking during waste collection were at lower risk for anti-HAV (+).

**Table 2:** Univariate analysis of Anti-HAV (+) among municipal workers.

Characteristic	Anti-HAV (+)	Anti-HAV (−)	*p* value
**Sex**	n/N, (%)	n/N, (%)	
male	72/138 (52%)	66/138 (48%)	<0.001^ b^
female	18/70 (26%)	52/70 (74%)	
**Age**^ a^	50.54 (7.4)	41.18 (6.9)	<0.001^ c^
**Duration of employment (years)** ^a^	18.12 (6.67)	13.41 (6.17)	0.001^ c^
**Occupation**			
Waste collectors	61/100 (61%)	39/100 (39%)	
Gardeners	29/108 (27%)	79/108 (73%)	<0.001^ b^
**Educational status**			
≤9 years	71/118 (60%)	47/118 (40%)	<0.001^ b^
>9 years	71/90 (79%)	19/90 (21%)	

^a^ mean, standard deviation. ^b^ X^2^ test. ^c^ Student’s *t*-test.

**Table 3:** Multivariate analysis of Anti-HΑV (+)among municipal workers (waste collectors *vs*. gardeners).

Variable	Odds Ratio (OR)	95% Confidence Interval (95% CI)
**Sex**		
male	1.00 (ref)	0.49–2.94
female	0.69	
**Age group**		
≤42 years	1.00 (ref)	7.29–69.88
>42 years	22.57	
**Occupation**		
Gardeners	1.00 (ref)	1.24–6.62
Waste collectors	2.87	
**Education group**		
>9 years	1.00 (ref)	1.01–4.78
≤9 years	2.19	
**Duration of employment**		
≤16 years	1.00 (ref)	1.15–11.08
≤16 years	3.57	

**Table 4:** Personal behavior during waste collection and HAV infection among waste collectors.

Variable	Anti-HAV (+)	Anti-HAV (–)	*p* value
Smoking/drinking/eating during waste collection	n/N, (%)	n/N, (%)	<0.001
Yes	49/61 (80%)	10/39 (74%)	
No	12/61 (20%)	29/39 (26%)	

## 5. Discussion

The prevalence of HAV infection among the municipal waste collectors under study was 61%. Logistic regression analysis indicated that age and occupational exposure of the worker are independently associated with the risk of an anti-HAV positive result; after adjustment made for sex, age, level of education and duration of employment In addition, the analysis of data restricted among the municipal waste collectors (n = 100) indicated that collectors who reported frequent exposure to smoking and drinking during waste collection procedures had a 1.84 fold increased risk of HBV infection in comparison to their colleagues who reported doing so seldom or never.

There are limited published studies about MSWWs’ risk of HAV infection. Corrao *et al.*, in a survey among 93 solid waste-workers in Asti, Italy, found no association between waste collection and risk of HAV infection [4]. Dounias and Rachiotis investigated the prevalence of HAV markers among 159 municipal employees (71 waste collectors; 88 office and blue collar employees without exposure to waste management) of a municipality in the broader region of Attica, Greece [5]. The prevalence of total anti-HAV antibodies (+) was significantly higher among waste collectors (62.5%) in comparison to the reference group (37.5%) Logistic regression analysis indicated that occupational exposure to waste was an independent risk factor for HAV infection. In another Greek study Mariolis and coworkers found a prevalence of 53.6% (anti-HAV IgG) among waste collectors in a municipality of the Attica region in Greece [6]. In addition, a study from Thailand has shown that the prevalence of HAV infection among public garbage collectors was 89.2%.Our study has some limitations which should be taken into account in the interpretation of the results. The first limitation is the cross-sectional design of the study, and therefore we are unable to attribute cause and effect. The assessment of exposure to work practices like smoking, drinking during waste collection process was based on self-reports and thus, some information bias may be expected. In addition, the result of the present study can not apply across the board of all municipal solid waste workers in Greece. Moreover, our results suggest a strong age-cohort effect on the prevalence and risk of HAV infection. (OR for age: 22.57). As it is well known, in the early 70s there was a significant prevalence of HAV infection in Greece, which declined dramatically in the 80s and 90s [9,10,11]. Last, although we have adjusted for education which remained a strong independent determinant of HAV infection in multivariate analysis, a residual confounding effect by education could not be excluded. However, in order to have a better assessment of the occupational exposure to waste—apart from job title—we have tried to assess possible work practices which could be associated with an activation of the faecal-oral route of HAV transmission among waste collectors (e.g., smoking/drinking/eating during waste collection). Moreover, our recent study on HBV infection among waste collectors indicated that exposure to improperly discharged needle sticks could be the way of transmission for HBV among waste collectors. On the contrary the present study has shown that poor working practices (e.g., smoking during waste collection) could be the transmission pathway for HAV infection.

In conclusion, the results of our study suggests that there is a significant prevalence of HAV infection for MSWWs indicating one possible pathway of virus transmission as being that of smoking or drinking during waste collection. Apart from vaccination against HAV, educational campaigns on good work and personal hygiene practices focused on municipal waste collectors could have further essential contribution to the control of HAV infection among members of this occupational group.
